# 1Comparative assessment of pediatric right ventricular volumes and function by MRI: right horizontal long axis versus short axis

**DOI:** 10.1186/1532-429X-15-S1-O102

**Published:** 2013-01-30

**Authors:** Brian D Soriano, Mark Ferguson, Johannes von Alvensleben, Seth Friedman, Randolph K Otto

**Affiliations:** 1 Cardiology, Seattle Children's Hospital, Seattle, WA, USA; 2 Radiology, Seattle Children's Hospital, Seattle, WA, USA; 3 Pediatrics, Seattle Children's Hospital, Seattle, WA, USA

## Background

Accurate and reproducible assessments of right ventricular (RV) size and function are important for clinical decisions. RV volume assessment by CMR has been traditionally performed using a short axis orientation (SAX). This projection, however, renders en-face views of the mitral and tricuspid valves. Distinction between atriums and ventricles can be difficult, which can complicate contour tracings for ventricular function. To improve reproducibility, alternative orientations for RV assessment have been proposed. This investigation sought to compare right horizontal long axis (RHLA) and SAX reproducibility in a pediatric cohort.

## Methods

**Figure 1:**
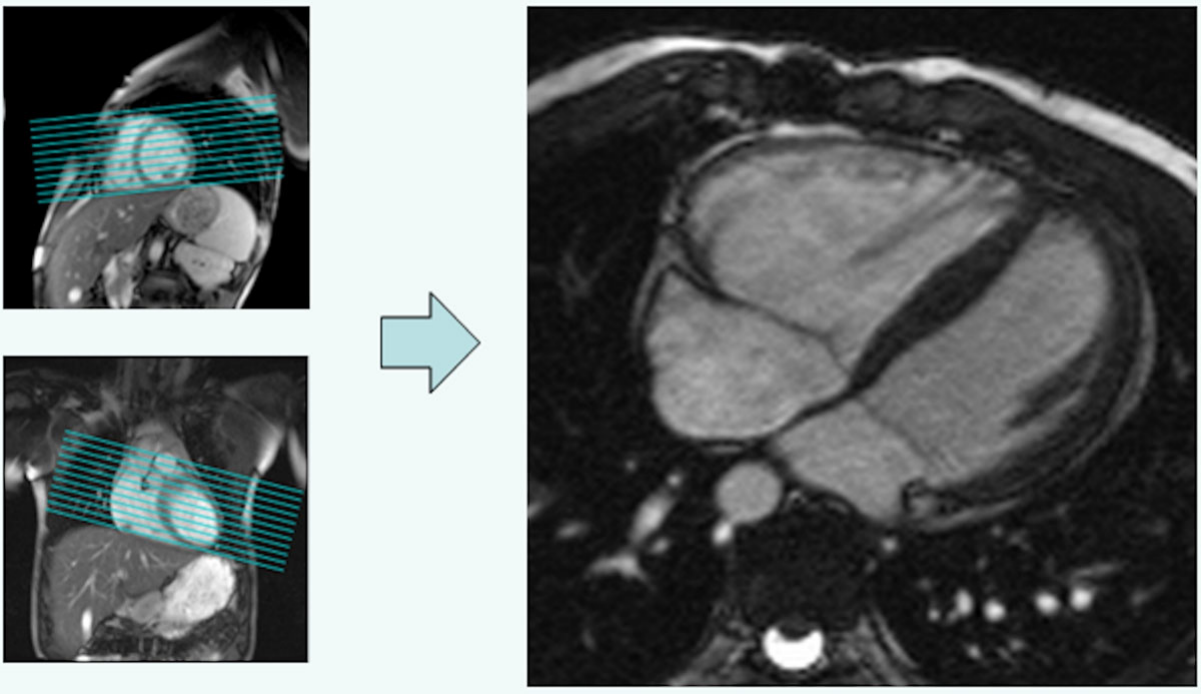
RHLA prescription (left) and resulting image plane (right).

The RHLA method is an oblique-transverse cine stack where the planes are oriented parallel to the diaphragmatic aspect of the right ventricle (Figure 1). We performed a retrospective evaluation of 20 CMR patients born with right heart disease (age range 7 years - 17 years), in which RHLA and SAX steady-state free precession cine stacks were originally obtained on all patients. Siemens' Leonardo software was utilized for contour tracing and volumetric calculations. Guidelines for contour tracing were reviewed by all readers to ensure comparable technique. Four separate readers participated in the study, with a wide range of experience. One of the 4 readers had no prior CMR experience, and practiced on 4 non-study patients while being trained by an attending. No further feedback was given until after study completion. The 4 readers, blinded to each others' results, measured RV end diastolic (EDV) and end systolic (ESV) volumes for every RHLA and SAX data set. In order to minimize confounders, no cross referencing with additional views was performed. All RHLA and SAX volumes were then re-measured by all readers, to determine inter-rater and intra-rater associations. Differences between mean volumes obtained with each orientation were also assessed.

## Results

Mean EDV among the 4 readers was 209.1 ± 65.4 ml by RHLA and 199.2 ± 62.0 by SAX (mean difference 9.9 ml, p=0.49). Mean ESV was 105.8 ± 45.5 ml by RHLA and 101.1 ± 46.3 by SAX (mean difference 4.7 ml, p=0.64). There was no significant difference between individual readers, however measurements by the least experienced person tended to be smaller than the others. RHLA EDV and ESV volumes tended to be 4-5% larger than those measured by SAX, even when factoring only the experienced readers. Mean ejection fractions by RHLA and by SAX were 49.2% and 48.9% respectively. Inter-rater reliability was high, and is reported in Table 1. Intra-rater reproducibility was high for all raters regardless of cardiac phase and image orientation (intraclass correlation coefficient range 0.94-0.97).

**Table 1:** Inter-rater reliability (n=20)

	Cronbach's alpha	Inter-item Correlation
RHLA EDV	0.991	0.966
SAX EDV	0.984	0.941
RHLA ESV	0.994	0.980
SAX ESV	0.986	0.952

## Conclusions

Both RHLA and SAX measurements result in high inter- and intra- rater reproducibilities, with no clearly-defined advantage between the two approaches. RHLA EDV and ESV volumes tend to be 4-5% larger than those measured by SAX. These differences should be considered when applied to clinical decision-making.

## Funding

None.

